# Sequence Analysis of Inducible Prophage phIS3501 Integrated into the Haemolysin II Gene of *Bacillus thuringiensis var israelensis* ATCC35646

**DOI:** 10.1155/2012/543286

**Published:** 2012-03-06

**Authors:** Bouziane Moumen, Christophe Nguen-The, Alexei Sorokin

**Affiliations:** ^1^UMR1319 Micalis, CRJ Institut National de la Recherche Agronomique, Bat. 440, Domaine de Vilvert, F-78352 Jouy-en-Josas, France; ^2^Sécurité et Qualité des Produits d'Origine Végétale, UMR408, INRA Université d'Avignon, 84914 Avignon Cedex 9, France

## Abstract

Diarrheic food poisoning by bacteria of the *Bacillus cereus* group is mostly due to several toxins encoded in the genomes. One of them, cytotoxin K, was recently identified as responsible for severe necrotic syndromes. Cytotoxin K is similar to a class of proteins encoded by genes usually annotated as haemolysin II (*hlyII*) in the majority of genomes of the *B. cereus* group. The partially sequenced genome of *Bacillus thuringiensis* var *israelensis* ATCC35646 contains several potentially induced prophages, one of them integrated into the *hlyII* gene. We determined the complete sequence and established the genomic organization of this prophage-designated phIS3501. During induction of excision of this prophage with mitomycin C, intact *hlyII* gene is formed, thus providing to cells a genetic ability to synthesize the active toxin. Therefore, this prophage, upon its excision, can be implicated in the regulation of synthesis of the active toxin and thus in the virulence of bacterial host. A generality of selection for such systems in bacterial pathogens is indicated by the similarity of this genetic arrangement to that of *Staphylococcus aureus* *β*-haemolysin.

## 1. Introduction

Many bacterial strains of the *B. cereus *group are pathogenic to different eukaryotic organisms, including animals, insects, and nematodes [1–6]. The caused illnesses are mainly attributed to the synthesis of toxins and protective cellular structures, usually encoded by plasmids or, in the cases of diarrheic food intoxications, in the chromosome. In addition to the importance of plasmids, carrying the toxins, it was also suggested that the temperate phages can be involved in the adaptation of these bacteria to animal hosts [1, 7]. It is indicative in this respect that several sequenced genomes of these bacteria possess large extrachromosomal elements encoding plasmid-related and phage-related functions [1, 5, 8–12]. The pathogen evolution can thus be regarded as a constant dynamic exchange of genes between plasmids and temperate phages integrated or not into the bacterial chromosome. A notable illustration of such prophage-plasmid coevolution is the similarity between the genome of a large phage 0305f8-36, isolated from *B. thuringiensis* and a contig of genome of the strain *B. weihenstephanensis *KBAB4 [13, 14]. In fact, this contig corresponds to the 417 kb extrachromosomal element pBWB401 and could therefore be regarded as either a plasmid or a nonintegrated prophage. Still no evidence exists for the association between phages and toxicity genes in the *B. cereus *group, although the presence in plasmids of genes for highly effective toxins, like that of anthrax, entomotoxic crystal protein, or emetic toxin, was well documented [4–6, 11, 15]. A recent report relates the evolution of temperate phages and the regulatory elements involved in adaptation of *B. anthracis* to the animal host [7].

A partial sequence of the *B. thuringiensis* var.* israelensis *ATCC35646 genome was deposited earlier as a “permanent draft” to the NCBI *Entrez* database [16]. The future use of these data would rather envisage looking for the answers to defined biological questions, instead of completing of the whole genome. In particular, our interest was related to the prophages integrated into this genome. We found during analysis of the ATCC35646 contigs, corresponding to 3,500–3,800 kb of the 5.4 Mb *B. cereus *ATCC14579 genome sequenced earlier [17], that several temperate phages were integrated in this area into the genome of ATCC35646. One of these prophages has appeared to be particularly interesting, since it was inserted into the gene *hlyII*, encoding haemolysin II, characterized in several *B*. *cereus* strains [18–21]. This toxin belongs to the same family of proteins as cytotoxin K, responsible for important cases of food poisoning [22–24]. The excision of such a prophage can lead to the formation of active toxin gene and thus, the prophage can be implicated in the toxin synthesis regulation. We therefore decided to identify the contigs and complete the sequencing of the region corresponding to this prophage. The completed prophage sequence permits to derive the genomic organization of this prophage. We also tested if the excision of the corresponding phage DNA can take place in this strain.

## 2. Results

### 2.1. Detection of Multiple Prophages in the *B. thuringiensis* ATCC35646 Genome and Nucleotide Sequence of phIS3501 Integrated into the hlyII Gene

The assembly of *B. thuringiensis* var* israelensis *ATCC35646 genome, deposited in NCBI *Entrez*, consisted of 866 contigs with accession numbers NZ_AAJM01000001 to NZ_AAJM01000866 [16]. The sequencing coverage was estimated to be ~6.2-fold and only contigs longer than 1500 bp were used for comparison to other genomes [16]. Like most bacteria of the *B. cereus *group, the genome of this strain encodes diarrheic toxin components: cytotoxin K (the gene* cytK*, locus RBTH0664, contig #1900), NheA (RBTH01882, contig #1388), NheB (RBTH01881, contig #1388), NheC (RBTH01258, contig #1255), HblC (RBTH03191, contig #1573), and HblA (RBTH03163, contig #1589). The gene encoding HblD must be located in the noncovered by sequencing regions. The paralogous to the *cytK* gene, *hlyII*, was found in this strain interrupted by a cluster of genes coding for several phage-related proteins. Two parts of this gene, N-terminal (locus RBTH03378) and C-terminal (RBTH01357), were separated on two contigs (##1604 and 1648, resp.). We found this situation interesting since the excision of this prophage should lead to the formation of the entire functionally active *hlyII* gene. This arrangement resembles sporulation *sigK* gene of *B. subtilis* [25–28] and Clostridia [29] and *β*-haemolysin gene *hlb* of *S. aureus *[30–32]. 

**Figure 1:**
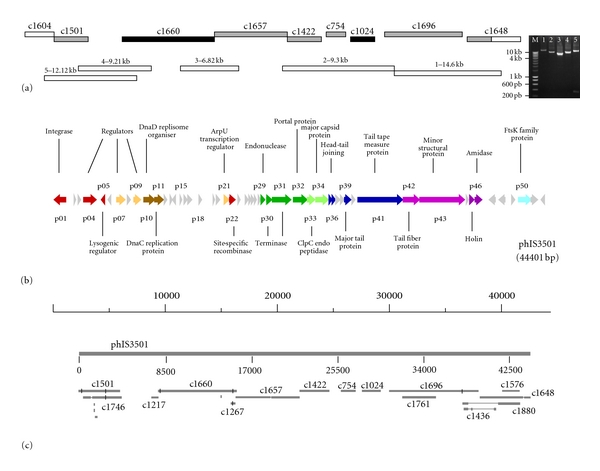
Analysis of the chromosomal region containing phIS3501. (a) Bars on the top show the sequencing contigs from GenBank entries NZ_AAJM01000001–NZ_AAJM01000866, corresponding to the region encoding phIS3501. Unfilled parts correspond to bacterial sequences, grey parts correspond to the phage contigs detected using *ERGOlight*, in black are the GenBank contigs assigned to this phage during the sequencing proceeds. The unfilled bars on the bottom show the PCR products seen on the gel to the right, used for the sequence assembly verification. The numbers indicate gel lanes and the expected product sizes, corresponding to the following primer couples: (1) PHISB8 × PHISE8; (2) PHISC3 × PHISE5; (3) PHISB3 × PHISD7; (4) PHISD6 × PHISG8; (5) PHISA1 × PHISH2. A few molecular weight marker sizes (lane M) are indicated to the left of the gel. (b) Sequence-based genetic map of phIS3501. Similarity- or position-based identification of gene functions are shown by short descriptions of encoded proteins. Predicted protein coding genes correspond to the *Entrez* sequence acc. # JQ062992. In grey are the genes with hypothetical functions. Other colours correspond to the functional phage modules: red (p01, p04, p05 and p22) and yellow (p07, p09 and p21), lysogeny and lysogenic regulation, brown (p10 and p11), replication; dark green (p29–p32)—DNA packaging and maturation; green, blue and purple (p33–p36, p39, and p41–p43)—head and tail structural module; violet (p45 and p46), host lysis; and cyan (p50) indicates the FtsK family protein, probably involved in lysogenic recombination. Scale bar is in bp. (c) Contigs from Genbank entries NZ_AAJM01000001–NZ_AAJM01000866 revealed by BLASTN search against the completed phIS3501 sequence. The names of significant contigs with identities 80 to 94% are shown in italics. Other contigs, with the names shown in bold, have the identity of 97–99% and correspond to the phage. The entire phage sequence is represented on the top as the grey scale bar in bp.

Finishing of the sequencing of this prophage was done as described in Methods. The completed phIS3501 genome has the size of 44,401 bp and G+C content of 34.9%. The Figure 1(a) represents experimental data that confirm the correctness of sequence assembly. For this purpose, we used LR PCR amplification with primers specific to several regions of the prophage. The coincidence of the lengths of amplified products with that predicted from nucleotide sequence indicated the correctness of the assembling. This verification was necessary since the assembly of the *B. thuringiensis *ATCC35646 genome, available from NCBI, contains other contigs with high similarity to phIS3501. This similarity reflects the existence of multiple similar prophages inserted into the host genome.

A total of 52 protein-coding genes, varying in size from 53 to 1344 amino acids, were identified as described in Methods. Figure 1(b) (see also Supplementary Table  1 available on line at doi: 10.1155/2012/543286) presents the functional map of phIS3501. Similarity search of the completed sequence of phIS3501 against the 866 contigs deposited in NCBI revealed 19 DNA-DNA hits with the scores less than *e* ^−13^, corresponding to complete contigs or their parts with identities on the nucleotide level ranging from 80 to 99%. Apart from eight contigs corresponding to the prophage phIS3501 and having the identity scores of 97 to 99%, the search revealed parts of 11 contigs scoring from 80 to 94% of identity (Figure 1(c)). These hits represent the regions of contigs corresponding to other similar prophages. In the same way, we tested similarities to other completely sequenced phages or prophages of the *B. cereus *group. These included the Gamma phage and four Lambda prophages of *B. anthracis *[33, 34], phBC6A51, and phBC6A52 of *B. cereus *ATCC14579^T^ [17] and also phBC391A1 and phBC391A2 of “*B. cytotoxicus*” NVH391–98 [35]. Although the set of phages used in this analysis was not exhaustive, it revealed 13 additional contigs corresponding to prophages (not shown). Six of the identified phage-related contigs, with scaffold numbers 1740, 1746, 1749, 1759, 1797, and 1824 in *Entrez*, contained integration-replication gene clusters. Several other contigs containing significant phage-related gene clusters were also detected. Thus four contigs (##1427, 1666, 1720 and a part of the mentioned above #1824) corresponded to the phage DNA-packaging gene clusters. Four others (##1436, 1576, 1601, and 1880) contained the lysis module genes. Although, because of sequence gaps in the available shotgun assembly, it was not possible to join together the different functional gene clusters, we concluded that at least six other similar prophages could be found in the genome of *B. thuringiensis *ATCC35646. An attempt to make such estimation using individual phage proteins did not appear to be successful due to ambiguity during interpretation (not shown). During the time that this paper was under preparation, a partial sequence of the strain *B. thuringiensis* IBL4222 isolated from a cat has appeared in NCBI Entrez database (accession identifier CM000759.1) in the form of 383 contigs. It appeared that this strain is in fact also *B. thuringiensis *var *israelensis* and five of the contigs corresponded to phIS3501 having higher than 99.9% identity to our prophage sequence (Supplementary Table  2). 

For 24 of all proteins encoded in phIS3501 (46% of total number), we have found significant similarity with proteins of known biochemical functions (Figure 1(b)). For six of these proteins, the precise biological function in phage development cannot be assigned. These are four proteins involved in phage development regulation (p04, p07, p21 and p29), integrase p22, and FtsK family protein p50. For 28 (54%) of encoded proteins we, cannot predict a function, although 24 of them share similarity with other phage proteins and therefore can be regarded as conserved. For some of these hypothetical proteins putative phage-related biological functions can be postulated based on their locations in the phage genome (Figure 1(b)). No similarity was detected for four (8%) of predicted proteins. The genome of phIS3501 also contains tRNA-Met gene (not shown) located about 200 bp upstream of the gene p01 for the lysogenic integrase. In this region, no genes-encoding proteins were predicted. This location is interesting since it can suggest the involvement of this tRNA gene in the regulation of integrase expression and thus of the prophage DNA integration or excision. 

Five functional modules or gene clusters can be recognized in the genomic organization of phIS3501. These are lysogeny and lysogenic regulation module, replication, DNA packaging and maturation, and head and tail structural module and lysis (Figure 1(b)). The modular organization of these genes can prove to be useful in postulating of biological functions for the hypothetical proteins [36]. It is interesting to mention that most of the similar phages of the *B. cereus *group contain in their genome an additional module, which includes the gene encoding for an ATP-dependent DNA transporter of FtsK family. The role of this protein in phage biology was not yet experimentally studied and is unclear at present. However, the vicinity of this gene, in the autonomously replicating phage DNA, to lysogenic integrase suggests that this module can be involved in phage lysogeny. The lysogeny and lysogenic regulation module of phIS3501 contains nine genes (p01–p09), the most evident functions are the integrase (p01) and the putative lysogenic repressor (p05). Integrase is the protein essential for the specific integration and, together with excisionase, which we cannot reliably recognize, excision of this phage. The above-mentioned *ftsK-*like gene can also be involved in this process, facilitating the integrase-mediated reaction. The lysogenic regulation involves also the repressor gene (p05) whose disruption would result in the clear-plaque phenotype.

In addition, the lysogenic regulation module includes also a helix-turn-helix protein and antirepressor (p04 and p07, resp.). The precise role of these genes in the regulation of similar phages of the* B. cereus *group was not yet experimentally studied. Based on its location, the tRNA-Met gene can also be involved in the lysogeny regulation. Insufficiency of sequence data on experimentally characterized excisionases from Gram-positive bacteria does not allow detecting by similarity search of the potential excisionase. Its function is needed to direct the integrase reaction out of the bacterial chromosome. Nevertheless, the appropriate localization and the similarity of size (85 aminoacids) and of content of positively charged aminoacids (15%) suggest that this could be the protein encoded by the p03 gene.

Another two regulatory proteins of this phage are encoded by p09 and p21. These are the RNA-polymerase sigma factor and ArpU-family regulator, the former could be involved in the regulation of late phage genes, as it was demonstrated for the Fah phage of *B. anthracis *[37], similar in its genomic organization to phIS3501. It is not clear, what is the exact role of the second integrase (p22), although it cannot be excluded that this protein is involved in the lysogenic conversion of the phage, participating in acquisition of functions useful for the bacterial host [7]. The replication module of phIS3501 contains two genes encoding DNA replication initiator (p11) and primosome loader (p10). These proteins are very characteristic for the temperate phages of the *B. cereus *group and their counterparts are found in most of the prophages residing in the sequenced genomes [5].

The phylogenic position of proteins encoded in the packaging module can indicate the type of DNA processing during the phage packaging. Large subunit of terminase in phIS3501 (p31) clusters with terminases characteristic to the phages this DNA maturation is driven by the recognition of *cos*sites [38]. The most similar protein from the phage genomes is that of *Geobacillus* phage E2. Highly similar are also the terminases of *B. subtilis *phage phi105 and of *B. anthracis *phages Cherry, Gamma, W*β* and Fah. The phIS3501 can therefore be positioned among the phages using the *cos*-type of DNA-packaging initiation.

The head and tail module genes of tailed phages define, in a great extent, the specificity of phages to their bacterial hosts. Although in the phage phIS3501, this module includes more than ten genes, only two of them, p42 and p43, are supposed to be directly involved in the bacterial host recognition. The orthologues of these two genes, encoding tail fiber protein and a minor structural protein, respectively, were shown to be involved in specific binding of similar phages from *B. anthracis *to the host [39]. Specific binding of the counterpart of p42 from the phage W*β* was experimentally demonstrated using fusion with the Green Fluorescent Protein (GFP). Involvement of the p43 counterpart was postulated due to finding of many mutations changing the phage specificity [39]. It was also shown that the host cell encoded protein GamR is one of essential components for binding of similar phages, presumably the phage receptor [40].

The lysis module genes, in particular the phage endolysin, were also shown to be highly related to the bacterial host specificity, suggesting their great usefulness for diagnostic and therapeutic purposes [41, 42]. It appeared that the p46 lysine gene of phIS3501 contained a frame shift in the fifth codon, thus leading to encode a nonfunctional protein (not shown). Therefore, the synthesized phage phIS3501 is not able to autonomously finish the lytic phage cycle, due to its inability to provide the lysis of bacterial host. However, since the bacterial genome encodes six or more of other similar prophages, it is possible that this function can be accomplished by one of them.

We concluded from the genomic sequence that the region encoding phIS3501 is able to provide autonomous phage DNA replication, excision, and synthesis of structural components, but it cannot provide the autonomous release of the phage particles, if they are formed, from the bacterial cells.

### 2.2. Induction of Lytic Development of Temperate Phages in *B. thuringiensis* ATCC35646

**Figure 2:**
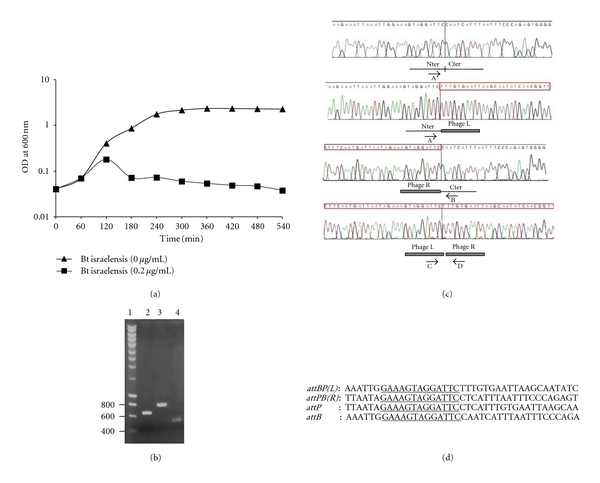
phIS3501 induction. (a) Effect of mitomycin C treatment on growth of *B. thuringiensis *var* israelensis* ATCC35646. Optical densities are shown of cultures treated (squares) or untreated (triangles) by mitomycin C (0.2 *μ*g/mL) added at time point of 60 min. (b) Detection of replicative form of phIS3501 DNA. PCR reactions were done using the total DNA extracted from the cell pellet (lanes 2 and 3) or supernatant (lane 4) 20 min after mitomycin C addition. Primers used: lane 2, PHISI2 × PHISI3 (specific to phage DNA); lane 3, PHIST6 × PHIST7 (specific to host chromosomal DNA); lane 4, PHISK6 × PHISS8 (specific to replicative or mature form of phage DNA). A few molecular weight marker sizes (lane 1) are indicated in bp to the left of gel. Obtained product sizes correspond to the ones expected from the sequence. (c) Sequencing tracks corresponding to integrated and excised DNA of phIS3501 and of the host. DNA for PCR amplification was extracted from noninduced overnight culture of *B. thuringiensis *var* israelensis *ATCC35646. Cartoons under the sequencing tracks show their interpretation in relevance to the phage integration status. Nter and Cter are the chromosomal parts of the *hlyII* gene. Phage L and Phage R are the ends of integrated phIS3501. The phage sequences are shown inside of red bar above the sequencing tracks. PCR amplification and (sequencing) primers were, from top to the bottom: PHISC7 × PHISE8 (PHISA2); PHISA2 × PHISC8 (PHISA2); PHISK5 × PHISC5 (PHISC5); PHISK6 × PHISS8 (PHISK6). (d) The bacterial and phage attachment sites. The derived phage (*attP*) and bacterial (*attB*) attachment sites based on the data presented in (c). Thirteen bases common sequence, presumably recognized by the phage integrase, is underlined.

The induction experiments reported here have the goal to show if the genomic region that we designated phIS3501 is able to be induced for self-replication and to be excised from the genome. Also, we have tried to test whether this sequence corresponds to an inducible prophage that can be detected by using a sensible bacterial host. The phage DNA was tested by PCR, using specific oligonucleotides, and the biological activity of the phage was tested using several strains of bacteria phylogenetically closely related to the strain *B. thuringiensis *ATCC35646. The mitomycin C induction experiment is represented on Figure 2(a). Total DNA was extracted from the supernatant of lysed bacterial culture and from the noncompletely lysed cells collected by centrifugation. PCR amplification experiments and sequencing of the corresponding products show that phIS3501 is able to induce the synthesis of the replicative form of its DNA, and to excise it from the host chromosome (Figure 2(b) and 2(c)). This form can be detected by PCR even in the cells nontreated with mitomycin C (Figure 2(c)). The excision of this prophage from the chromosome results in formation of the whole-length *hlyII *gene, thus enabling the cells to produce this toxin.

Twenty strains, closely related to *B. thuringiensis *ATCC35646, from recently described collection [43], were tested for the formation of phage-related plaques. However, we did not succeed in finding any sensitive host to be used as indicator (data not shown). This is surprising since, as reported above, the strain *B. thuringiensis *ATCC35646 contains at least seven different prophages in the chromosome and also possesses the nonintegrated linear phage-like element, presumably also corresponding to a phage [16]. We consider this result as not definitive and rather due to our ignorance of proper conditions for obtaining the plaques for these phages and hosts.

Analysis of sequences of the circularized form of phage DNA and its integration site in the bacterial chromosome permitted us to identify the 13 bp sequence common in the phage DNA and in the chromosome (Figure 2(d)). This 13 bp consensus sequence is presumably recognized by the site-specific phage integrase p01.

## 3. Discussion

Many bacterial sequencing projects do not result in establishing the completed sequences of studied genomes. The relative amount of such data will certainly increase with the advent of extrahigh throughput methods. The reason is that, even if the finished data represent much higher value than the “skimmed” ones, the so-called finishing is a laborious enterprise, containing a significant amount of manual work that can hardly be automated. Comparative utility of both approaches was discussed [44–47] but it is certain that the investment in precise experimental data acquisition requires case-to-case justification and cannot be generalized. The work that we present here uses previously assembled shotgun data deposited into public databases [16]. The initial complete assembly of the 40 kb long prophage was hampered by insufficiency of experimental data and also by the complexity that was due to existence of highly similar, but different, sequences in the same genome. We completed the sequence of this prophage that permitted us to understand the extent of its integrity. For this purpose, we used the similarity of organization of such prophages to those already entirely sequenced. This revealed the potential sequencing contigs covering this region and thus minimized the subsequent combinatorial PCR experiment. Since we detected also the existence of at least six other similar prophages in this genome, the independent verification of the final assembly of this area was applied. Finally, our complete prophage assembly was also confirmed by independent shot-gun sequencing and assembly of a strain *B. thuringiensis* IBL4222, which appeared to be another sequenced *israelensis* strain (NCBI accession identifier CM000759.1).

This prophage was chosen to be entirely sequenced since it is integrated into an important haemolysin gene. The availability of completed prophage sequence permitted us to make preliminary conclusions of its functionality and to test it experimentally. We demonstrated the ability of this prophage to induce its DNA excision and ligation. However, since the endolysin gene of the prophage is interrupted by internal frameshift, we cannot expect formation of the mature phage particles without functional involvement of other induced prophages. The excision of this sequence from the bacterial chromosome leads to the formation of uninterrupted *hlyII *gene, encoding a potentially active haemolysin. We did not, however, find the experimental conditions to demonstrate the synthesis of this protein (not shown).

Two other intensively studied systems can be mentioned that represent a similar mode of gene regulation by creating of uninterrupted coding sequence upon a temperate phage excision. The first is the well-known *skin *element, inserted into the coding sequence of *sigK *gene of *B. subtilis* [25–28]. Genomic sequencing revealed that the *skin *element is in fact a prophage that lost many functions essential for formation of phage particles [48]. Actually, the *skin*-dependent regulation of this sigma-factor synthesis is not essential for sporulation of *B. subtilis *and such insertions do not exist in other *Bacillus* species [28, 49, 50]. However, such a construction is important for the correct sporulation timing in Clostridia [29]. Another remarkable example of gene regulation by prophage excision is the haemolysin gene of *Staphylococcus aureus*. The phage *ϕ*NM3 is integrated into the *β*-haemolysin gene (*hlb*) of many *S. aureus *strains [30–32]. Since *ϕ*NM3 carries several virulence factors, its induction not only leads to the formation of active haemolysin gene but also increases its physiological effects by weakening the host immune system. Surprisingly, the endolysin gene in this prophage is also mutated [31, 51]. In the case of the prophage phIS3501, described here, we did not detect any obvious virulence factor associated with the prophage. However, since six other similar prophages are integrated in the host genome, some of them, being simultaneously induced, may provide such factors.

At present, the involvement of *hlyII *gene of the *B. cereus *group bacteria in virulence for animals is not entirely clear. Several papers report that this system can be rather important. A pathogenicity-related protein of this family, cytotoxin K, was described in the remote strain “*B. cytotoxicus*” NVH391–98 and considered as the main factor responsible for toxicity in a case of collective food poisoning [22]. The toxicity was regarded as to be related to a particular allele of this gene, the others do not seem to be so hazardous due to the weakness of gene expression level and cell lysis ability of the protein [23, 24, 52]. Two other papers demonstrate the increase of virulence of bacterial cells due to synthesis of the *hlyII* gene product [53, 54].

The similarity of the arrangement of prophage phIS3501 associated with the *hlyII* gene to the one of *S. aureus *phage *ϕ*NM3 integrated into the *β*-haemolysin gene may indicate a general character of selection for such systems. The fact that a similar genetic arrangement is found for *sigK *of such remote organisms as *B. subtilis *and *C. difficile* and for haemolysins of *S. aureus *and *B. thuringiensis *indicates that common evolutionary advantages could exist. Therefore the functionality of phIS3501 merits further investigation especially with relatedness to the virulence, if any. An important factor would be development of an experimental system for measuring of pathogenicity of the strain *B thuringiensis *ATCC35646 or similar in relation to the haemolysin II gene functioning.

## 4. Conclusions

The complete nucleotide sequence and genomic organization of the prophage phIS3501 inserted into the *hlyII *gene of *B. thuringiensis *var* israelensis *ATCC35646 was established. The prophage encoded lysine gene contains a frameshift that can prevent formation of phage particles. Nevertheless, the concerted cell lysis that we observed applying mild mitomycin concentrations could be due to induction of one of other six prophages residing elsewhere in the host genome. Excision of phIS3501 from bacterial chromosome leads to formation of uninterrupted *hlyII *gene, encoding a potentially active haemolysin. A general character of selection for such systems in bacterial pathogens is indicated by the similarity of this gene arrangement to that of *Staphylococcus aureus β*-haemolysin.

## 5. Methods

### 5.1. Bacterial Strains, Growth Conditions and DNA Manipulations

The strain *Bacillus thuringiensis *var* israelensis *ATCC35646, the same that was used for the genomic sequencing [16], was obtained from Dr. Alla Lapidus (*Integrated Genomics*). Bacterial growth, total DNA preparation, and PCR conditions were as described [43, 55].

### 5.2. Sequencing of the phIS3501 Genome

To determine the entire sequence of the prophage phIS3501, we used the data of shotgun assembly, available from the *ERGOlight *database (http://www.ergolight.com/ERGO/), deposited to NCBI *Entrez* under the accession numbers NZ_AAJM01000001–NZ_AAJM01000866 (866 contigs). The contig #1648, containing a part of the gene* hlyII*, interrupted by the phage-encoding DNA, was tested, using the *Pinned Regions *tool implemented in the *ERGOlight *database, for colinearity with other *B. cereus *group genomes containing similar prophages elsewhere in the chromosome. This procedure, interactively applied, permitted to detect five contigs (##1501, 1657, 1422, 754 and 1696 in *ERGOlight*) presumably corresponding to the phIS3501 prophage. Contiguity of these regions in the bacterial chromosome, together with the contigs #1604 and 1648, containing parts of the *hlyII *gene, was verified by Long Range PCR (LR PCR, Figure 1). Thus, the sequencing substrates were generated which were used to complete the sequence of the entire phIS3501 by primer walking. For that 129 oligonucleotides and 416 additional sequencing runs of 700 bases of average length were produced. Xbap or Gap4 software [56, 57] was used for the sequence assembly. The average coverage of the *de novo* sequenced regions was approximately 7-fold. The completed assembly of phIS3501, once again confirmed by LR PCR, revealed two additional contigs (#1660 and 1024) from the *ERGOlight *corresponding to this prophage. Supplementary Table  3 presents the oligonucleotides, used for sequence assembly confirmation and in phage induction experiments and their positions in relation to the phage. The *de novo *annotated sequence of phIS3501 was deposited to NCBI *Entrez* under the accession number JQ062992.

### 5.3. Phage Induction

The overnight culture of *B. thuringiensis *var *israelensis* ATCC35646 grown at 37°C in liquid medium under aeration (200 rpm/min) was diluted 100-fold and incubated in the same conditions. Mitomycin C was added after 1 h to the final concentration 0.2 *μ*g/mL. This concentration was found in pilot tests (not shown) to be the minimal needed to induce the prophages without causing the inhibition of bacterial growth. Culture supernatant or total cellular DNA extracted from 1 mL of precipitated by centrifugation cells were used for PCR analysis of the presence of excised phage DNA.

### 5.4. Phage Genome Analysis and Gene Predictions

The consensus sequence of phI3501 genome, generated by the assembly software, was used for gene prediction by GeneMark program [58] implemented at web site of the Georgia Institute of Technology (http://opal.biology.gatech.edu/GeneMark/). The start positions of predicted genes were manually scrutinized and corrected, if a better potential Ribosome Binding Site was detected, using the Sequin NCBI tool (http://www.ncbi.nlm.nih.gov/Sequin/). Search for homology of phage-encoded proteins to the NCBI database was done using BLAST tools implemented there or at the LIRMM (Le Laboratoire d'Informatique, de Robotique et de Microélectronique de Montpellier) web-site (http://www.phylogeny.fr/) [59]. The ACLAME database (http://aclame.ulb.ac.be/) was used to detect the similarity with bacterial virus proteins [60]. The gene functions were assigned using similarity to known proteins and also taking into account the positions of genes in phage genomes [36]. tRNAscan-SE [61] was used for tRNA prediction.
